# A Ten-Year Trend of Cutaneous Leishmaniasis at University of Gondar Hospital, Northwest Ethiopia: 2009-2018

**DOI:** 10.1155/2021/8860056

**Published:** 2021-03-09

**Authors:** Ayalew Jejaw Zeleke, Adane Derso, Arega Yeshanew, Rezika Mohammed, Helina Fikre

**Affiliations:** ^1^Department of Medical Parasitology, School of Biomedical and Laboratory Sciences, College of Medicine and Health Sciences, University of Gondar, Gondar, Ethiopia; ^2^Leishimaniasis Research and Treatment Centre, University of Gondar Hospital, Gondar, Ethiopia; ^3^Department of Internal Medicine, School of Medicine, College of Medicine and Health Sciences, University of Gondar, Gondar, Ethiopia

## Abstract

**Background:**

Cutaneous leishmaniasis (CL), which is one form of leishmaniasis, may show variations over years across regions, and epidemiological studies are crucial to estimate the cases of the disease status over a long time. This study is aimed at determining the trend of CL among patients at the University of Gondar Leishmaniasis Research and Treatment Center, northwest Ethiopia between 2009 and 2018 years.

**Methods:**

A ten-year data were extracted retrospectively. We included all patients who were visiting the center for CL diagnosis during the last ten years. Giemsa-stained skin slit microscopy was used to diagnose the disease. A chi-square test was used to compare the proportions of patients infected across years, seasons, months, sex, and age groups.

**Result:**

During the 10 years, a total of 1079 samples were requested for the diagnosis of CL. The cumulative average annual prevalence was found to be 55.4% (598/1079). The highest and lowest proportions of CL cases were recorded in 2014 (69.5%) and 2018 (35.4%), respectively. However, the percentage of CL cases did not show any significant differences over the study period. The number of suspected patients was significantly increased over the years (being lowest in 2009 and highest in 2017). The proportion of CL cases showed a remarkable difference across months but not seasons. CL was the highest within 15-49 years of age and males.

**Conclusion:**

The prevalence of CL did not show any significant differences over the last ten years. However, a remarkable increase of CL suspected cases was observed across the years. The disease showed significant association with age, sex, and months, but not seasons.

## 1. Background

Leishmaniasis is one of the neglected tropical diseases (NTDs) caused by different species of leishmania, an obligate intracellular protozoan parasite. The disease is endemic in 98 countries, particularly localized in areas of the tropics and subtropics of Africa, in settings ranging from rain forests in America to deserts in western Asia [1]. It occurs in both the New and Old Worlds [2]. There are different forms of leishmaniasis—visceral, cutaneous, and mucocutaneous [3]. Cutaneous leishmaniasis (CL) is the most common form of leishmaniasis [4]. Countries with higher cases of CL include Middle East, Southeast Asia, South America, and Africa including Ethiopia [5]. In sub-Saharan Africa, *L. donovani* is the only species causing visceral leishmaniasis (VL) [6], and CL is caused by *L. major*, *L. tropica*, *L. aethiopica*, or *L. donovani* [7].

Cutaneous leishmaniasis is damaging socially and deeply stigmatizing people [8]. Social stigma, prejudice, marginalization, and the extreme poverty of affected populations are among the factors contributing to the neglect of these diseases. Lack of funding for the prevention and treatment of these diseases is also a contributing factor [9]. The increased risk of infection is mediated through poor housing and environmental sanitation, lack of personal protective measures, and economically driven migration and employment that bring nonimmune hosts into contact with infected sand flies [10].

Cutaneous leishmaniasis is transmitted by female phlebotomine sand fly bite. *Phlebotomus orientalis* is the predominant species, which transmit the disease in both the highland and lowland areas of Ethiopia. The vector regurgitates promastigote form of the parasite and will be ingested by skin macrophages to transform amastigotes. These amastigotes cause disease in humans and affect cellular immunity [11]. In Ethiopia, the disease is mainly caused by *Leishmania aethiopica* [12]. CL presents as a nodule on an exposed part of the body and later becomes widely disseminated as skin lesions of various forms (wet and/or dry ulcer). The lesions may persist for years without any tendency to healing, resistant to treatment, and abundant parasites in skin smears. It can cause life-long scars and serious disability [4]. Most of the Ethiopian cases resemble lepromatous or dimorphic forms [13]. Cutaneous leishmaniasis-related disabilities impose a great social burden, especially on women, and reduce economic productivity [14].

In Ethiopia, CL has been reported since the 1900s [15]. In spite of its long recognized endemicity, it remains largely invisible, and information on the magnitude of CL are still insufficient both nationally and at regional levels, however, the annual incidence is estimated from 20,000 to 50,000 cases [16, 17]. This is mainly because most of the affected patients hide themselves as they are from remote areas. It is also due to the social stigma associated with the deformities and disfiguring scars caused by the disease and negative perceptions and attitudes towards treatment options [18].

Implementation of CL control and prevention methods is challenging due to the presence of multiple reservoir hosts and complexity of its vector control [19]. A guideline for the management of CL was produced in 2013 in Ethiopia. However, only a few health care facilities provide the service. Both clinical assessment and microscopic examination of the parasite from skin lesion have been used to diagnose CL. Antimonials are approved for CL treatment, but most cases are treated traditionally [17]. In Ethiopia, a variety of medicinal plants are used as natural medicines without a scientific base. Plant extracts or plant-derived compounds provide an important source of new medicinal agents [20, 21]. Medicinal plants used by traditional healers in different locality of the country are *Euphorbia abyssinica* local name (“Kulkual”), *Englerinawoodfordioides* (“Teketsila”), *Bruceaantidysenterica*J (“Abalo”) [22], *Phytolacca dodecandra* (“Endode”), *Gossypium spp.* (“Jirbi”) [23], *Sphearanthus steetzii* (“Qoricha –Cheffe”) [24], and *Clematis simensis* (“Hazo”) [25].

Regardless of the guideline development, to date, there is no effective leishmaniasis vector control program. Distribution of insecticide-treated nets (ITNs) and insecticide spraying for malaria control may have some impact on phlebotomines in lowland localities where VL is also endemic. In general, there is inadequate evidence, nor control efforts of CL in the country [26]. Therefore, substantial works are needed for the prevention and control of CL.

Epidemiological data such as trends of CL positivity rates at health care facilities is essential to plan suitable interventions. In addition, there is no published data about the trend of CL cases in the country in general and at Gondar leishmaniasis research and treatment center in particular. Therefore, the present study is aimed at assessing the CL positivity rate for the past 10 years at the center as a proxy measure for the trend of CL in the area, and it may contribute to evidence-based decision on the disease control activities.

## 2. Method and Materials

### 2.1. Study Design, Area, and Period

A cross-sectional retrospective study was conducted at the University of Gondar Hospital Leishmania research and treatment center. The center is found in Gondar, which is located in the Central Gondar Zone of the Amhara Regional State of Ethiopia. Gondar is found in the North of Lake Tana and Southwest of the Simien Mountain. It has a latitude of 12°36′N 37°28′E with an elevation of 2133 meters above sea level. It is 727 km far from Addis Ababa which is the capital city of Ethiopia [27]. Patients, whose laboratory result was eligible and with full information, were included in the study. The data were collected in March 2019.

### 2.2. Data Collection and Laboratory Method

Ten years (2009-2018) laboratory registered data regarding CL were extracted. Both negative and positive test results for CL suspected cases were included in the study. Microscopy is the tool used for the diagnosis of CL. Information regarding the patient's age, sex, month, and year of examination were collected using a case report form.

### 2.3. Data Analysis and Interpretation

Data were extracted from laboratory log books and summarized using Microsoft Excel. Then, data are entered and analyzed using SPSS 20 software package. Sequence chart analysis was used to evaluate trends of the data. A Pearson's Chi-square test was used to describe the trend and association of variables.

## 3. Result

### 3.1. Trend of CL over a Ten-Year Period (2009-2018)

**Figure 1:**
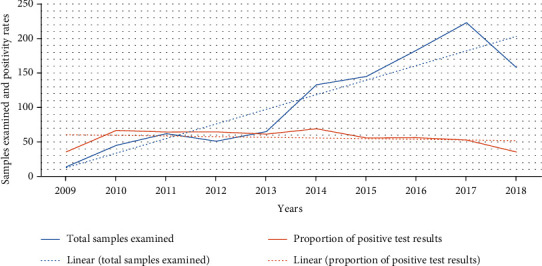
Trend for CL suspected and proportion confirmed cases in LRTC at University of Gondar, Northwest Ethiopia, 2009-2018.

During the last 10 years, a total of 1079 samples were requested for CL diagnosis. Of these, 598 (55.4%) were diagnosis with CL. The highest (69.2%) and lowest (35.4%) proportions of CL were recorded in 2014 and 2018, respectively. However, the prevalence rate of CL did not show significant differences across the study years. On the other hand, there was a significant increase of CL suspected cases over the last ten years, from 14 in 2009 to 223 in 2017 (Figure 1).

### 3.2. Skin Slit Test Results Stratified by Age and Sex

The age of the patients ranged from one month to 88 years, with a mean age of 28.72 years (standard deviation 15.39 years); 65.1% of them were male. CL showed a significant association with age. The number of cases was the highest among the 15-49 years of age category followed by 49 years and above; the lowest cases were seen among the under fifteen years of age groups. Moreover, the positivity rate was higher (65%) among males than females (37%), and the difference was statistically significant (Table 1).

### 3.3. Monthly and Seasonal Patterns of CL over the Last Ten Years

**Figure 2:**
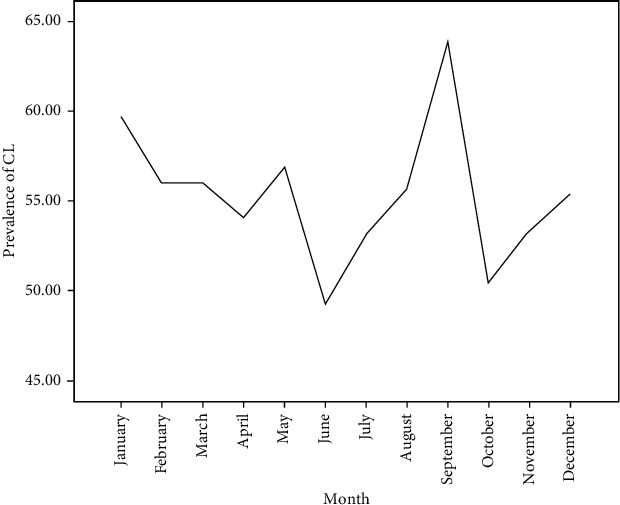
The aggregate monthly prevalence rate of CL cases at LRTC, University of Gondar, Ethiopia, 2009-2018.

Over the last ten years, the aggregate monthly prevalence rate of CL showed a significant fluctuation, and the highest prevalence rate (63.8%) was reported in September, followed by January (59.7%) and May (56.9%); the least percentage (49.3%) was reported in June (Figure 2).

**Figure 3:**
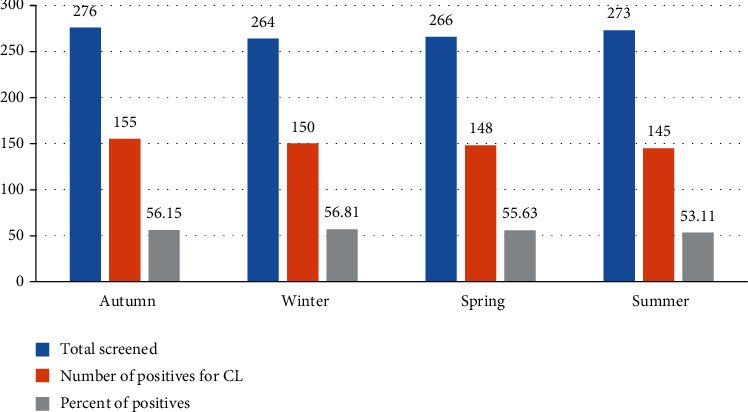
Distribution CL suspected and confirmed cases among different seasons at LRTC, University of Gondar, Ethiopia (2009-2018).

The prevalence rate of CL in relation to seasons ranged from 53.11 to 56.15%. However, there was no significant variation across the seasons (Figure 3).

## 4. Discussion

This study could be used as a proxy to estimate the spread and impact of CL across similar settings of Ethiopia. According to an electronically based database search, the prevalence of the disease has not yet been fully addressed in different areas of the country. Thus, the current study has provided information about the trend of CL cases over ten years among patients who seek treatment at the Leishmaniasis Research and Treatment Center (LRTC), University of Gondar Hospital, northwest Ethiopia.

Over the last 10 years, the overall positivity rate of CL at the center was 55.4%. Unfortunately, its positivity rate did not show any significant difference across the years. This may indicate that the existing prevention and control measures are not effective enough in reducing the cases of the disease. In Ethiopia, CL diagnosis and treatment services are given only in a few health care facilities, and most are available in areas far from places where the actual patients are residing [28]. This could have complicated the prevention and control of the disease. As a result, the disease transmission remained stable over a long time. Thus, the current study implies the necessity of epidemiological surveillance of the disease in order to design and implement appropriate interventions in CL endemic areas of the country.

In comparison to other studies, the average cumulative annual positivity rate of CL in this study is higher than that of a study conducted in Addis Ababa, Alert hospital (14.2%), a three-year retrospective study, which included 1651 leishmaniasis suspected cases, and found 234 positive cases [29]. The variation might be related to differences in duration of the study period and area. Moreover, when we observe the distribution of confirmed CL positive cases, 96 of positive cases were from Addis Ababa, the capital city of Ethiopia [30] and comparably a city where better socioeconomic condition and many health facilities are existing, and the remaining positive cases came from different regions of the country. On the other hand, almost all patients in the current study are from rural and more endemic areas which are well-known for sand fly replication in Ethiopia [31, 32].

The present study showed that the number of CL suspected cases stood at 14 in 2009 and significantly increased to 223 in 2017 (Figure 1). The increasing number of suspected cases might be related to an increase in health-seeking behavior of the people. A few studies in this study area indicated that people believe that the disease is caused by bats, and others related the disease to lack of hygiene and punishment from God [28]. Previously, due to the limited availability of antileishmanial drugs, there was a trend to prioritize VL. This might have created a bias in the community that CL will not be treated unless severe or complicated form of the disease is developed. Moreover, a recent study on CL clinical features and treatment response in the present study area indicated that 53.9% had received treatment for their CL episode prior to presenting at the Leishmania Research and Treatment Center (LRTC), most often traditional medicine 28.6% [33]. Currently, the Ethiopian national guideline on leishmaniasis control and other studies indicated the need of further studies on the efficacy of the antileishmanial drugs so as to increases treatment response and enable increment in patients flow to health institution [33, 34].

This study also showed that the prevalence of CL infection is higher among the 15-49 years of age group than the other age categories (Table 1). This finding is in agreement with those of studies conducted in Addis Ababa, Ethiopia [29], and Al Hassa, Saudi Arabia, Kashan city in Iran [35]. This might be due to the fact that this age group is the most productive and often engaged in agricultural activities in the fields, and this may coincide with the exophilic behavior of sand fly vectors in these endemic areas [12, 36]. It has also been observed that CL is significantly higher (65%) in men than women (37%). This result is similar to those of studies done in Al-Munawarah Province, Saudi Arabia, Hamadan Province, west Iran, and Kashan city central Iran [37–39]. Unlike women who spend their time indoors, men are engaged in agricultural activities and are more exposed to the bite of the sand fly vector infected with the parasite.

In Ethiopia, especially in rural areas, most activities are grouped either for man or woman. Men are usually engaged with outdoor activities like farming, keeping cattle, staying around gorges, and/or farmland for a long period. On the other hand, women are expected to cook food, fetch water, and raise children. This job discrepancy may lead men prone to contact with the habitat of the sand fly and acquisition of the disease [15, 40]. Furthermore, the gender difference in CL incidence is attributable to sex hormonal effects or immune responses [41], as it has been noted in some other parasitic diseases [42]. Due to the exophilic behavior of *P. longipes* and *P. pedifer*, humans are bitten during the day time when they visit hyrax habitats, even if they visit human dwellings at night and return to their outdoor resting sites [36, 43].

In our study, CL prevalence is higher in the month of September followed by January over the ten years of the twelve months (Figure 2). In northern Ethiopia, ploughing, seeding, and weeding of crops are done at the end of June and in the early of July of every year. Thus, farmers could be exposed to sand fly bites in such periods. Generally, the parasite requires two months for clinical incubation time. Hence, this positivity rate might be correlated with the parasite life cycle. In other words, individuals who are infected in early July may develop the most common clinical features in September. Similarly, November is the major harvesting month; therefore, people infected in this particular month may lead to develop CL in January, and such people might have gone to health facilities during this time for screening. This finding is in line with that of a study conducted in Al Hassa, Saudi Arabia, and Southwest of Iran [35, 44].

## 5. Limitation

As it was secondary data, it was not able to collect other essential variables such as environmental conditions, socioeconomic status, demographic, and human behaviors. The result should therefore be interpreted with due consideration of the limitation.

## 6. Conclusion

The prevalence of CL did not show any significant differences over the last ten years. On the other hand, there was a remarkable increase in CL suspected cases over the years. This finding may have several implications for disease control. First, the effect of any control measures was insignificant in the study area as this study revealed that the prevalence remain stable over the years. Thus, a close monitoring of the control strategies and their effectiveness will be needed in the future. Second, the information about the significant association of CL with age, sex, and month can be used for planning of interventions. Efforts, for example, may be effective if they are focused on males and the most affected age groups.

## Figures and Tables

**Table 1:** Age- and sex-related patterns of CL prevalence in LRTC, University of Gondar, Northwest Ethiopia (2009-2018).

Variables	CL positive (%)	CL negative (%)	Chi-square test	*P* value
Sex				
Male	458 (65.2)	244 (34.8)	78.429	0.000
Female	140 (37.1)	237 (62.9)		
Age in years				
<15	54 (32.1)	114 (67.9)	54.04	0.000
15-49	486 (61.8)	300 (38.2)		
>49	58 (46.4)	67 (53.6)		

## Data Availability

All data generated or analyzed during this study are included in this published article.

## Abbreviations

CL: Cutaneous leishmaniasis
LRTC: Leishmania Resaerch and Treatment Center
VL: Visceral leishmaniasis
WHO: World Health Organization.
